# New Approach to Determine the Activity Concentration Index in Cements, Fly Ashes, and Slags on the Basis of Their Chemical Composition

**DOI:** 10.3390/ma16072677

**Published:** 2023-03-28

**Authors:** Andrés Caño, José Antonio Suárez-Navarro, Francisca Puertas, Ana Fernández-Jiménez, María del Mar Alonso

**Affiliations:** 1 Eduardo Torroja Institute for Construction Sciences (IETcc-CSIC), 28033 Madrid, Spain; 2 Environmental Radioactivity and Radiological Monitoring Unit (URAyVR), Centro de Investigaciones Energéticas, Medioambientales y Tecnológicas (CIEMAT), Avda Complutense, 40, 28040 Madrid, Spain

**Keywords:** cement, fly ash, slag, NORM, activity concentration index, HJ-Biplot

## Abstract

The manufacture of Portland cement entails high energy and environmental costs, and various solutions have been implemented in recent years to mitigate this negative impact. These solutions include improvements in the manufacture of cement clinker or the use of supplementary cementitious materials (SCMs), such as fly ash (FA) or slag as a replacement for a portion of the clinker in cement. The incorporation of these SCMs in cement may increase its radiological content as they are naturally occurring radioactive materials (NORMs). The Activity Concentration Index (ACI) is a screening tool established in the European EURATOM Directive 2013/59 to determine the radiation protection suitability of a final construction material. The ACI is determined by the activity concentrations of ^226^Ra, ^232^Th and ^40^K, usually determined by gamma spectrometry. The methodology of gamma spectrometry is accurate and appropriate, but this technique is not available in all laboratories. For this reason, and taking into account that there is a relationship between the chemical and radiological composition of these building materials, a new approach is proposed to determine the radiological content of these materials from a chemical analysis such as X-ray fluorescence (XRF). In this paper, principal component analysis (PCA) is used to establish the relationships between the chemical composition and radiological content of cements, FAs, and slags of different natures. Through PCA it was possible to group the cements based on two variables: CaO content and Fe_2_O_3–_Al_2_O_3–_TiO_2_ content. A lower correlation was observed for the FAs and slags, as the sample scores were centered around the origin of the coordinates and showed greater dispersion than the cements. The clusters obtained in the HJ–Biplots allowed the determination, using multiple regression, of models relating the activity concentration of ^226^Ra, ^232^Th (^212^Pb), and ^40^K to the oxide percentages obtained for the three matrices studied. The models were validated using five cements, one FA and one slag with relative percentage deviations (RSD(%)) equal to or less than 30% for 89% of the activity concentrations and 100% of the ACI determined.

## 1. Introduction

The Activity Concentration Index (ACI) is a screening value, introduced in European Directive EURATOM 2013/59 [1], used to determine the suitability of building materials from the point of view of radiological protection [2,3,4]. It is determined by the following expression (1):

(1) $$\mathrm{ACI}=\frac{C_{226_{\mathrm{Ra}}}}{300}+\frac{C_{232_{\mathrm{Th}}}}{200}+\frac{C_{40_{K}}}{3000}$$

where $C_{226_{\mathrm{Ra}}}$, $C_{232_{\mathrm{Th}}}$, and $C_{40_{K}}$ are the activity concentrations of ^226^Ra, ^232^Th, and ^40^K. For the building material to comply with this Directive, the ACI value must be ≤1 mSv y^−1^, not taking into account the environmental background. These activity concentrations can be determined by various methodologies, the most common being gamma spectrometry due to the fact that it is a nondestructive technique that allows the activity concentration of these radionuclides to be determined via a single measurement [5]. The effective dose is calculated from the absorbed dose for a model based on a room measuring 2.8 m × 4 m × 5 m with a wall width of 20 cm and a density of 2.35 g cm^−3^ [6].

Article 75 of EURATOM 2013/59 refers to the materials for which the external radiation dose from gamma radiation must be guaranteed, and therefore, the ACI must be known. This list includes final construction materials and those listed in Annex XIII to the aforementioned Directive. These materials are industrial waste such as Fly ash (FA), phosphogypsum, phosphorus slag, tin, copper, red mud (from aluminum production), and steel production waste and all of them are naturally occurring radioactive materials (NORMs).

Portland cement concrete, which has Portland cement as the main binder, is the most versatile and widely used building material in the world. The clinker for this Portland cement is manufactured from a mixture of two main components—limestones and clays—which undergo clinkering at temperatures between 1450 and 1500 °C. Both limestones and clays have minor components other than SiO_2_, CaO, Al_2_O_3_, and Fe_2_O_3_ that become part of minor phases of Portland cement clinker [7]. The importance of the raw materials used in clinkerization is the key factor for the final composition of the clinker phases and will therefore determine the properties of the final material [8,9,10]. This clinker, together with a setting regulator (usually a hydrated or anhydrous calcium sulfate) gives rise to Type-I Portland cement (as defined in EN 197-1:2011 [11]).

However, there are some other types of noncommon cements, not covered in the EN 197-1:2011 standard [11], which may be affected by changes in raw materials or the production process are as follows:

- White cements, according to the UNE 80117-2012 standard [12] are those that have a whiteness CIELAB not lower than 85. These cements are also defined in the UNE 80305 standard [13] and are characterized by having a low content of Fe_2_O_3_ and TiO_2_.
- Belite cements (BCs) contain mainly C_2_S, C_3_S and calcium aluminates and produce high amounts of C–S–H gel, which leads to very good durable properties [14,15].
- Calcium aluminate cements (CAC) [16,17,18,19,20], are manufactured from limestones and bauxites. Their main oxides are CaO and Al_2_O_3_, with a variable content of Fe_2_O_3_, TiO_2_, and MnO. The main phases of CAC are monocalcium aluminate (CaAl_2_O_4_), mayenite (C_12_A_7_), belite (C_2_A), gehlenite (C_2_AS), etc.
- Calcium sulfoaluminate cements (CSA), have as main phases ye’elimite ($C_{4}A_{3}\overset{-}{S}$) and belite ($C_{2}S$). A feature to highlight in this type of cement is that they have a low carbon footprint [21,22,23].

The high energy and environmental costs associated with the cement manufacturing process [24] have led to the use of different measures to mitigate these negative effects. One of these measures is the use of new starting materials in the preparation of the cement raw mix, such as ceramic waste [25], incinerator ash [26], or crystallized slag [25]. Another measure is to use alternative fuels in the clinkering kilns, such as sewage sludge, animal meal, or agricultural residues [27,28]. The use of alternative raw materials or fuels inevitably produces changes in the chemistry and mineralogy of the clinker and its potential final reactivity.

However, one of the most effective means of mitigating the environmental effects associated with the manufacture of cement is the use of supplementary cementitious materials (SCMs) to partially replace clinker and thus lead cement manufacture towards higher levels of sustainability and lower greenhouse gas (GHG) emissions. Although these SCMs are usually chemically reactive waste from other industries, in many cases they can be NORMs. Cements with different contents and proportions of these SCMs account for 26 of the 27 cements included in the EN 197-1:2011 standard and comprise types II, III, IV and V. Both CEM I and those containing some type of SCM must comply with the requirements laid down in EURATOM 2013/59.

Meanwhile, among the SCMs used in cement manufacture, FA from coal-fired power plants and slag from blast furnaces are the most widely employed.

FA is an industrial by-product produced from the combustion of pulverized coal in thermal power plants at temperatures up to 1600 °C, which generates amorphous, spherical particles. FA is composed mainly of silica (SiO_2_), alumina (Al_2_O_3_), iron oxide (Fe_2_O_3_), and calcium oxide (CaO) [29].

Under ASTM C618 [30], FA is denominated F-type siliceous if it has a CaO content of less than 10%. These ashes are typically produced from the combustion of anthracite or bituminous coal and the sum of the SiO_2_, Al_2_O_3_, and Fe_2_O_3_ content typically represents 70% of the ash’s composition. Type C or calcareous FA [30], on the other hand, has CaO content greater than 10% and is produced from the combustion of lignite or sub-bituminous coal. The sum of the main oxide content (SiO_2_, Al_2_O_3_, and Fe_2_O_3_) is between 50 and 70% [31,32].

Raw coals typically contain mineral impurities, such as clays, quartz, feldspars, and shales, which can remain in suspension and become part of the FA via solidification during the cooling process.

It is known that the performance of FA as an SCM depends on its chemical and physical characteristics, which in turn depend on the chemical and mineralogical composition of the feed coal, the degree of pulverization, the combustion conditions, the temperatures employed, the ash collection methods, etc. [32,33]. However, although much research has been conducted on the presence of natural radionuclides in FA, there is no evidence of a relationship between such radioactivity and the chemical composition and characteristics of FA [34,35,36,37,38,39,40,41]. Only Kovacs et al. [42] stated that the concentration of natural radionuclides in FA is due to the amount of sulfur and heavy metals in the coal. However, in a previous study, the authors observed a dependency between chemical composition and the content of naturally occurring radionuclides based on the type of coal burned [43].

Ground granulated blast-furnace slag (GGBS) is a by-product of the manufacture of iron and steel from a mixture of iron ore, coke, and limestone. The slag is the result of the fusion of the acid gangue of the iron ore material and the sulfur ashes of the coke with the lime and magnesium of the limestone used as melting flux at temperatures of 1500–1600 °C [44,45]. GGBS is mostly amorphous with some mineralogical phases detected by XRD, such as melilite (solid solution of gehlenite 2CaO-Al_2_O_3_-SiO_2_-akermanite 2CaO-MgO_2_-SiO_2_) and merwinite (3CaO-MgO-2SiO_2_). The composition of the slag depends on the starting iron material, the steelmaking process, and the flux materials used, which can include natural materials such as limestone and dolomite. All these variables, affect both its chemical composition and its radiological content. The major oxides present, in rank order, are CaO, SiO_2_, Al_2_O_3_, and MgO, with the latter varying between 0 and 19% [46]. There is no literature linking the chemical composition or physical and chemical processes of GGBS formation with the natural radionuclide content. Other types of slag, such as those from steel or nickel production, are less commonly used as SCMs but are a potential option that should be further investigated.

Taking into account all of the above, it appears clear that the raw materials used in the manufacture of various cements—FA, and slags—could influence the presence of different natural radionuclides, and could thus be related to their chemical composition. Therefore, knowledge of the relationship between radionuclides from natural radioactive series (uranium, actinium, and thorium) and ^40^K and the chemical composition of cement, FA, and slags would allow for a new approach to estimating the activity concentration of these radionuclides and, thus, to determining the ACI of various cementing materials.

Three matrices were selected to individually study the cement itself and the two most common additions, FA and slags [31,47,48]. These materials are included in Annex XIII to EURATOM 2013/59, so knowledge of their ACI is necessary.

As previously stated, our working hypothesis is that the ACI can be estimated by understanding the relationship between the chemical compositions and activity concentrations of the natural radioactive series (uranium, thorium, and actinium) and ^40^K in ordinary Portland cement (OPC), calcium aluminate cement (CAC), calcium sulfoaluminate cement (CSA), FA, and slags. To test our hypothesis, correlations between the chemical compositions, obtained through X-ray fluorescence (XRF), and radioactive content, obtained through gamma spectrometry, were sought using principal component analysis (PCA) and HJ–Biplots based on previous studies [7]. Subsequently, we determined which models identified a relationship between the different quantities using multivariable linear regression with a backward stepwise approach. Finally, the models were validated for the activity concentrations of ^226^Ra, ^232^Th(^212^Pb), and ^40^K, and for the ACIs obtained from these activity concentrations.

## 2. Materials and Methods

### 2.1. Samples

The samples used in this study were of three types:

(a) 23 cements of different typologies:
  - 12 Type-I cements (samples C1, C4, C5, C6, C18, C20, C21, C23, and C25);
  - 2 white cements (WC) with a very low Fe_2_O_3_ content and no TiO_2_; (samples C11 and C12);
  - 3 sulfate-resistant cements, with a low proportion of tricalcium aluminate (samples C10, C24 and C26);
  - 2 belitic cements (C17 and C19) blended with FA;
  - 1 blended cement (CEM II/A-L) with a maximum of 20% limestone (sample C7);
  - 1 calcium sulfoaluminate cement with a low SiO_2_ content but a high sulfate proportion (sample C16); and
  - 5 calcium aluminate cements (CAC) whose main component is monocalcium aluminate (CA) (samples C13–C15, C22 and C27).
(b) 26 samples of (FA) with a CaO content of less than 10%, referred to as Type F according to ASTM C618 [30], from different thermoelectric power plants with different compositions and proportions of burned material (samples FA1–FA26).
(c) 17 samples of slags of different typologies: 13 of them are blast-furnace vitreous slag (samples S1–S3, S6–S8, and S10–S16), 3 steel slag samples (samples S4, S5 and S17) and 1 slag sample from nickel manufacture (S9).

The samples come from very different geographical areas in order to test whether the geographical origin of the samples and ultimately their raw materials, interfered with the proposed models.

### 2.2. Chemical Analysis of the Cement, Fly Ash, and Slag Samples

The chemical composition of the samples was determined by XRF on a Bruker (Billerica, MA, USA) S8 Tiger analyzer. Loss on ignition (LoI) was determined according to European standard EN 196-2:2014 [49]. In a previously published paper by the same authors [50], the chemical composition of 12 of the cements analyzed, as well as 11 FAs and 5 slags, were determined as follows:

- C1, C4–C7, C10–C16.
- FA1–FA11.
- S1–S5.

### 2.3. Determination of the Activity Concentration of the Radionuclides in the Samples

The activity concentration of the radionuclides present in the samples was determined by gamma spectrometry. For the measurements, the samples were placed in powder form in cylindrical plastic boxes (76 mm diameter, 33 mm height), which were sealed to avoid loss of ^222^Rn and left to stand for 21 days to allow equilibrium to be reached between ^226^Ra and its short-lived progeny. Samples and backgrounds were measured for 80,000 s and 600,000 s, respectively.

Three coaxial detectors were used: (1) Broad Energy (BEGe), (2) Reverse Electrode (ReGe) and (3) Extended Range (Xtra). The electronics associated with the detectors were of the Canberra (Canberra Industries, Meriden, CT, USA) brand and consisted of a high-voltage source, amplifier, analog-to-digital converter and AIM module to link the electronics chain to the PC. The three detectors were characterized by Canberra (Canberra Industries, Meriden, CT, USA) and the counting efficiency was calculated by LabSOCS following the method described in [5]. The spectra were acquired and analyzed using the Genie 2000 software [51].

The radionuclides determined in this study [52] were as follows: ^234^Th (63.30 (2) keV–3.75 (8)%), ^226^Ra (186.211 (13) keV–3.555 (19)%), ^214^Pb (351.932 (2) keV–35.60 (7)%), ^214^Bi (609.312 (7) keV–45.49 (19)%; 1120.287 (10) keV–14.91 (3)%; 1764.494 (14) keV–15.31 (5)%), ^210^Pb (46.539 (1) keV–4.252 (40)%), ^212^Pb (238.632 (2) keV–43.6 (5)%), ^208^Tl (583.187 (2) keV–85.0 (3)%, ^228^Ac (911.196 (6) keV–26.2 (8)%), ^235^U (163.356 (3) keV–5.08 (3)%; 205.16 (4) keV–5.02 (3)%; 143.767 (3) keV–10.94 (6)%), ^40^K (1460.822 (6) keV–10.55 (11)%), ^137^Cs (661.657 (3) keV–84.99 (20)%), and ^241^Am (59.5409 (1)–35.92 (17)%. Correction of spectral interferences due to ^235^U in the 186 keV photopeak of ^226^Ra and ^228^Ac in 1460 keV photopeak of ^40^K were corrected using the algorithm developed in [53]. Coincidence sum correction was performed using Genie 2000 and the peak-to-total method using the total efficiency curve provided by Canberra in the detector characterization.

Gamma spectrometry was determined in a previous study [50] for the same 12 cements, 11 ashes and 5 slags as those listed in Section 2.2.

### 2.4. Model Relating ^226^Ra, ^232^Th(^212^Pb), and ^40^K Activity Concentrations to Chemical Composition

**Figure 1:**
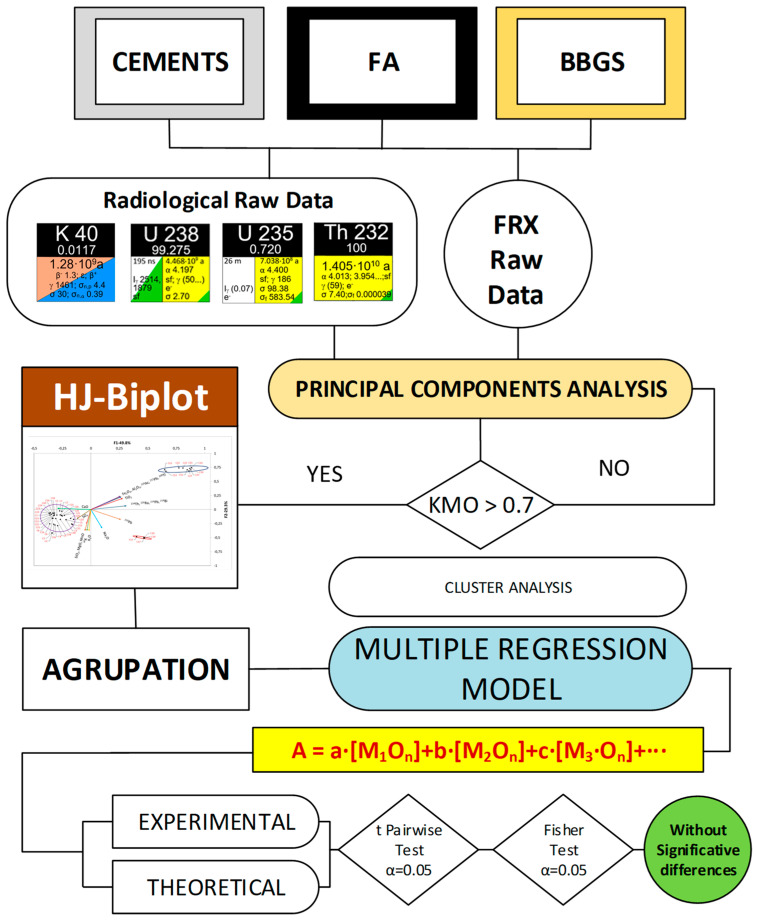
Diagram of the statistical process applied to the activity concentrations and chemical compositions obtained by XRF.

Based on the results of the chemical analysis of the samples and the activity concentration data of the radionuclides, a statistical study was carried out in order to obtain the models relating to both sets of results. The study was performed using the Real Statistics Resource Pack, Release 7.6 software (www.real-statistics.com; accessed on 1 August 2022) [54] and was carried out separately for the three sample types: cement, FA and slag. The diagram showing the statistical process followed in this study is presented in Figure 1.

The oxide percentage was determined according to the procedure described in Section 2.2 and the activity concentrations of the radionuclides belonging to the radioactive series of uranium, actinium, and thorium together with ^40^K were determined as described in Section 2.3.

The two sets of results were analyzed by PCA. The method used was to iteratively eliminate variables that did not allow a Kaiser–Meyer–Olkin (KMO) fitness index value higher than 0.7 to be reached [55]. The proposed KMO value ensured a satisfactory correlation between the sets of variables sampled [56]. The results obtained for the variables and the scores obtained from the samples were plotted on an HJ–Biplot [57]. The eigenvectors were orthogonally rotated using the Varimax procedure as it maximizes the variance of each component and allows a better interpretation of the results obtained [58]. The correlations between the variables or vectors were determined by the cosine of the angle they form with each other in the HJ–Biplot [59]. The sample scores were obtained by normalizing the input values by multiplying them by the factors obtained from the eigenvalues and eigenvectors in the PCA [60]. Clusters of the data were obtained by cluster analysis of the sample scores. The clusters obtained were plotted on the HJ–Biplot from the confidence ellipses.

The models used to relate the activity concentrations of ^226^Ra, ^232^Th (^212^Pb), and ^40^K to the percentages of the oxides obtained by XRF were performed by backward stepwise multiple regression analysis. This procedure allows for the elimination of variables that do not reach a significance level α below 0.05. However, this process did not eliminate the possible collinearity of the studied variables that could distort the obtained model [61]. For this reason, the variance inflation factor (VIF) of the variables in the model was calculated and its influence was verified for those values in which the VIF was higher than 10 [62].

### 2.5. Validation of the ACI Determined Using the Proposed Model

To validate the proposed model for each type of sample, 5 new cements (1 OPC CEM I 42.5R, 2 white cements (BL I 52.5R and BL II/B-LL 42.5R) with limestone addition, and two CACs) [47,63,64], one FA and one slag sample [43] were used afterwards. The samples’ chemical composition as determined by XRF is presented in Tables S1–S3 of the Supplementary Information.

The activity concentrations of ^226^Ra, ^232^Th(^212^Pb), and ^40^K obtained using the proposed models with the chemical compositions were compared with the experimental values of the cements, FA and slag using Student’s *t*-test of paired results for a significance level α = 0.05.

The estimated ACIs using ^226^Ra, ^212^Pb, and ^40^K calculated from the proposed models were compared with those obtained with their activity concentrations determined by gamma spectrometry using the relative standard deviation (RSD(%)). The acceptance criterion was that the RSD(%) should be less than or equal to 30%.

## 3. Results

### 3.1. Activity Concentrations and Chemical Compositions Obtained for CEMENTS, FA and Blast-Furnace Slag

The chemical compositions and activity concentrations of the radionuclides of the natural radioactive series of uranium, actinium, and thorium together with the ^40^K of the cements, FA and slag, are given in Tables S1–S6 of the Supplementary Information, as well as in the abovementioned study [50]. Table S7 shows the activity concentrations obtained in the previous paper for samples S3 and S4 where it can be seen that they are the only samples in which the presence of ^241^Am was detected. The activity concentrations of ^241^Am in slags S2 and S3 were 3.21 ± 0.26 Bq kg^−1^ and 3.92 ± 0.30 Bq kg^−1^, respectively.

**Figure 2:**
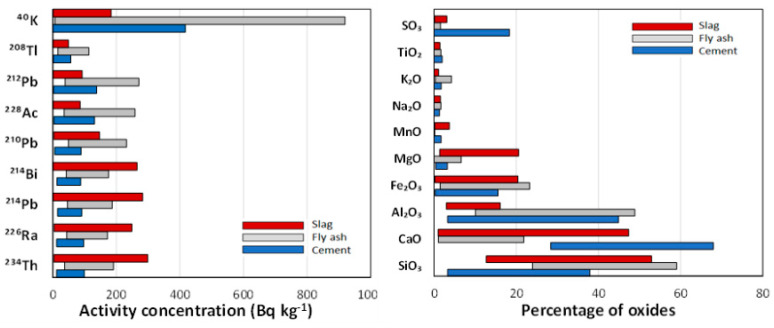
Activity concentration ranges of the radionuclides belonging to the natural radioactive series of uranium (^234^Th, ^226^Ra, ^214^Pb, ^214^Bi, and ^210^Pb) and thorium (^228^Ac, ^212^Pb, and ^208^Tl) together with the ^40^K, and chemical composition (in percentage of oxides) for the cements, FA and S analyzed in this study. The actinium (^235^U) series was not represented as all activity concentrations were below the lower limit of detection (LoD).

Figure 2 shows the oxide percentage and activity concentration ranges of the natural radioactive series of uranium (^234^Th, ^226^Ra, ^214^Pb, ^214^Bi, and ^210^Pb) and thorium (^228^Ac, ^212^Pb, and ^208^Tl) together with ^40^K. The actinium series (^235^U) was not represented in Figure 2 as all activity concentrations were below the lower limit of detection (LoD).

With respect to the chemical composition of the samples, the CaO percentage in cements is higher than in FA and slag, as expected [65]. The Al_2_O_3_ range is equivalent in FA and cements but lower in slag. The MgO range is higher in slag, especially in blast-furnace slag or GGBS, than in the different cements and FA. Finally, the SO_3_ range is higher in cements due to the CaSO_4_ in their composition. The remaining oxides can be considered equivalent in the three matrices studied.

The activity concentration range of ^234^Th, ^226^Ra, ^214^Pb, and ^214^Bi (corresponding to the radioactive uranium series) shows slag > FA > cements. With respect to the ranges of the radionuclides of the thorium series (^228^Ac, ^212^Pb, and ^208^Tl), these are higher for the FA and equivalent for the cement and slag. Finally, the ^40^K range is higher in the FA than in the cement or slag.

The cements showed a secular balance in the uranium and thorium series, with the thorium series ranking higher than the uranium series. The FA also showed a higher range of activity concentrations of the thorium series than the uranium series. Likewise, the activity concentration range of ^210^Pb was higher than that of the remaining radionuclides of the uranium series (^234^Th, ^226^Ra, ^214^Pb, and ^214^Bi). In the case of slag, a higher range of uranium series activity concentration was observed than for thorium. The behavior of ^210^Pb was opposite to that of the FA, as it was lower than that of the other uranium series radionuclides.

### 3.2. Relationships between Natural Radionuclides and Chemical Composition in Cement Samples

**Figure 3:**
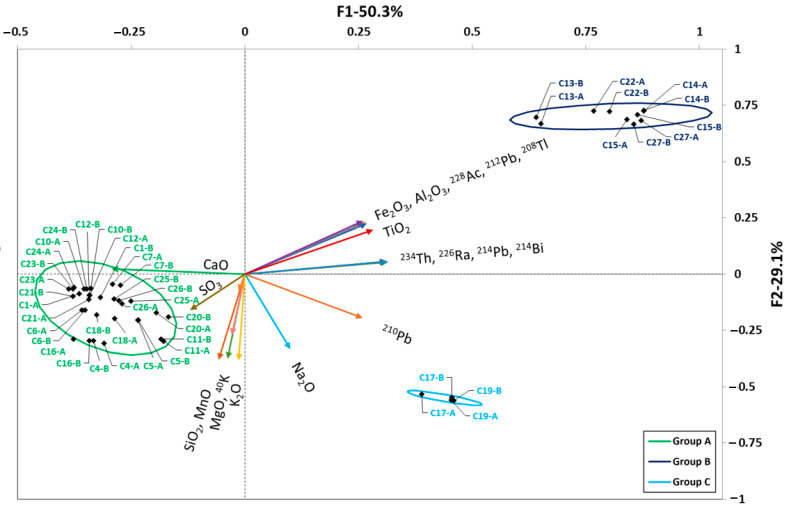
HJ–Biplot for the cements analyzed. The variables studied were CaO, SO_3_, SiO_2_, MnO, MgO, K_2_O, Na_2_O, TiO_2_, Fe_2_O_3_, Al_2_O_3_, ^234^Th, ^228^Ac, ^226^Ra, ^214^Bi, ^214^Pb, ^212^Pb, ^210^Pb, ^208^Tl, and ^40^K, represented as a vector. The scores of the cement samples were calculated from the weights of the studied variables. The cements were grouped into three sets according to their content: (a) CaO and SO_3_; (b) Fe_2_O_3_, Al_2_O_3_, TiO_2_, thorium series (^228^Ac, ^212^Pb, and ^208^Tl) and uranium series (^234^Th, ^226^Ra, ^214^Pb, and ^214^Bi); and (c) Na_2_O and ^210^Pb. The correlations between the studied variables were established from the cosine of the angle they form between them. The groupings were established by means of confidence ellipses determined at a significance level α = 0.05.

The HJ–Biplot obtained for the cements studied is presented in Figure 3. The KMO coefficient was 0.809, which indicated adequate data sampling [56]. The two factors found represented 79.4% of the variance of the factors analyzed.

The first factor, which represented 50.9% of the variance, was related to the amount of Fe_2_O_3_, Al_2_O_3_, TiO_2_, CaO, SO_3_, and radionuclides of the uranium series (^234^Th, ^226^Ra, ^214^Pb, ^210^Pb, and ^214^Pb) and thorium series (^228^Ac, ^212^Pb, and ^208^Tl). The second factor, which represented 29.1% of the variance, was related to the amount of SiO_2_, MnO, MgO, K_2_O and ^40^K.

The HJ–Biplot shows that increasing the Fe_2_O_3_, Al_2_O_3_, and TiO_2_ content in the cements increased the activity concentration of the radionuclides of the uranium and thorium radioactive series. On the other hand, increasing the CaO and SO_3_ content decreased the activity concentration of these radionuclides. It was also observed that ^210^Pb behaved differently than the other members of the uranium radioactive series.

Cluster analysis identified three clusters or groups which were delimited by confidence ellipses. The most abundant group (in green) was the one corresponding to Type-I cements, Type-II cements with limestone addition, white cements (with low Fe_2_O_3_ content and no TiO_2_), and calcium sulfoaluminate cement (C16), which had the lowest natural radionuclide content and the highest CaO percentage.

The second set included the CACs (samples C13–C15, C22 and C27) with a higher concentration of natural radionuclide activity and higher Fe_2_O_3_, Al_2_O_3_, and TiO_2_ content (dark blue group).

The C17 and C19 cements were characterized by higher amounts of SiO_2_, K_2_O, Na_2_O, and natural radionuclides in both the uranium and thorium radioactive series and ^40^K, thereby forming a third group. This third group was formed independently, as it contained two belite cements with a 30% FA addition, hence their low CaO content (light blue group).

### 3.3. Relationships between Natural Radionuclides and Chemical Composition in FA Samples

**Figure 4:**
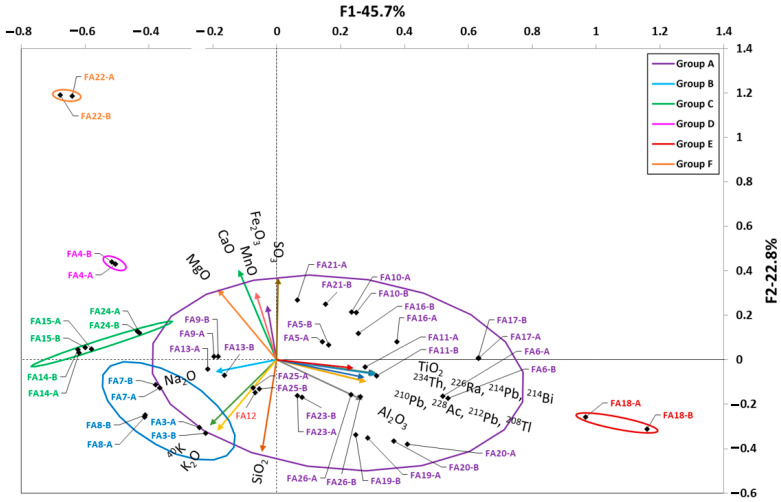
HJ–Biplot for the analyzed FA. The variables studied were CaO, SO_3_, SiO_2_, MnO, MgO, K_2_O, Na_2_O, TiO_2_, Fe_2_O_3_, Al_2_O_3_, radionuclides of the uranium and thorium series, and ^40^K, represented as a vector. The 6 groups obtained are represented by their confidence ellipses and show high variability as they are distributed in the 4 quadrants. FA22 can be considered an outlier with respect to the other 5 sets of samples.

Figure 4 shows the HJ–Biplot relating the chemical compositions of the FA samples to the activity concentrations of radionuclides belonging to the natural radioactive series of uranium and thorium together with ^40^K. The two factors found in the FA accounted for 67.6% of the variance of the analyzed samples. The KMO fitness index was 0.709, reaching an acceptable level of correlation between the sampled values. The first factor represented the amount of TiO_2_, Al_2_O_3_, Na_2_O, and radionuclides from the uranium and thorium radioactive series, explaining 45.7% of the variance. The second factor represented Fe_2_O_3_, SO_3_, MnO, CaO, MgO, SiO_2_, K_2_O, and ^40^K, explaining 22.8% of the variance. The scores obtained for the FA samples indicated a high dispersion as (i) they were distributed in the four quadrants and were close to the origin of the axes and (ii) six groups of samples were formed, three of which are very close to each other.

The most abundant group (Group A) is presented in purple and contains 14 FAs. Groups B (light blue; FA3, FA7, and FA8) and C (green; FA14, FA15, and FA24) each comprise three different FAs. Finally, three groups of only one FA each are established:

- Group D (pink FA4). This FA exhibits differentiated behavior along with Group C (green), formed by FA14, FA15, and FA24, all of them with below-average Al_2_O_3_ contents and higher CaO proportions than the other FAs.
- Group E (red FA18), which has a very high Al_2_O_3_ content, forms in itself an independent group and was the highest-scoring in the uranium and thorium natural radioactive series.
- Group F (orange FA22). The score for FA22 was further away from the other FAs due to its higher CaO content and lower SiO_2_ content. This F22 sample was eliminated from the final study to determine the statistical model as it is a thermal power plant landfill ash, but it confirms the interpretive power of the HJ–Biplots.

### 3.4. Relationships between Natural Radionuclides and Chemical Composition in Slag Samples

**Figure 5:**
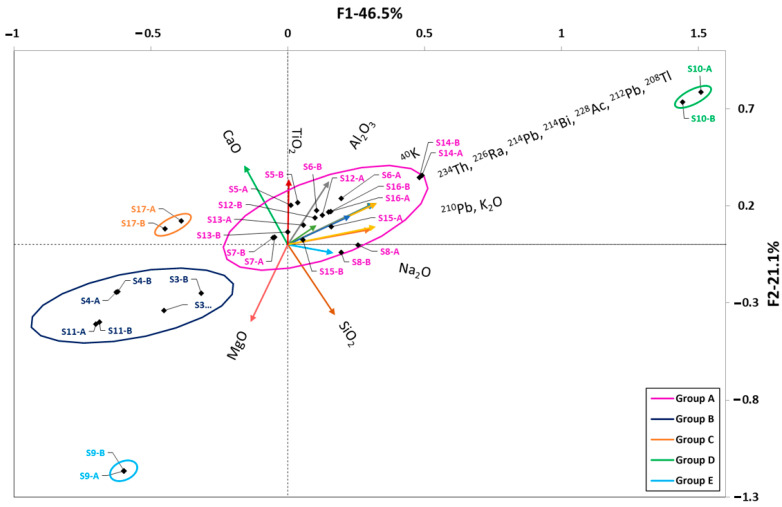
HJ–Biplot for the S analyzed. The variables studied were CaO, SiO_2_, MgO, K_2_O, Na_2_O, TiO_2_, Al_2_O_3_, radionuclides of the uranium and thorium series, and ^40^K, represented as a vector. The KMO suitability index value obtained was 0.700. Most of the sample scores are centered on the origin of the coordinates, reflecting a lower correlation with the two factors found. Slags S9, which is strongly correlated with MgO, and S10, whose high uranium and thorium series radioisotope content distinguishes them from the rest of the samples analyzed, are the only exceptions.

Figure 5 shows the HJ–Biplot relating the chemical compositions of the slag samples to the activity concentrations of radionuclides belonging to the natural radioactive series of uranium and thorium together with ^40^K. The two factors were found to account for 67.6% of the variance of the sampled values. The KMO fitness factor obtained was 0.700, which was set as the required correlation level for the PCA. A KMO value higher than 0.7 was obtained after eliminating MnO, SO_3_, and Fe_2_O_3_ from the variables studied.

Although these oxides are highly characteristic of slags [44], no direct relationship was found between them and the activity concentrations of the different radionuclides.

Factor 1, which represented 46.5% of the variance, was related to the natural radionuclide content (uranium and thorium series together with ^40^K), K_2_O, and Na_2_O. Factor 2, which represented 21.1% of the variance, was related to the Al_2_O_3_, TiO_2_, CaO, MgO, and SiO_2_ content. The clusters obtained in the sample scores indicated a low correlation with the factors found, as they were centered around the origin of the coordinates.

In this case, five groups (A–E) were established, three of which include only one slag:

- Group A (pink): The largest set of slags in the center of the graph were scored on the basis of their higher or lower uranium and thorium series radionuclide content.
- Group B (dark blue): S3, S4, and S11. These slags with a higher proportion of MgO or MnO had the lowest radioactive content when compared to the other slags.
- Group C (light blue): S9. This set had a composition more similar to the Group B slags but differed from the rest because of its higher MgO (20.60%) and SiO_2_ (52.92%) content and lower Al_2_O_3_ (2.98%) content. TiO_2_ was not present.
- Group D (green): Sample S10 contained the highest score in the uranium series (with a mean ^226^Ra activity concentration of 240.5 Bq kg^−1^).
- Group E (orange): S17 had a lower natural radionuclide content than the other samples.

### 3.5. Models Relating the Activity Concentration of ^226^Ra, ^232^Th(^212^Pb), and ^40^K to the Chemical Composition of the Cement, FA and Slag Samples

**Figure 6:**
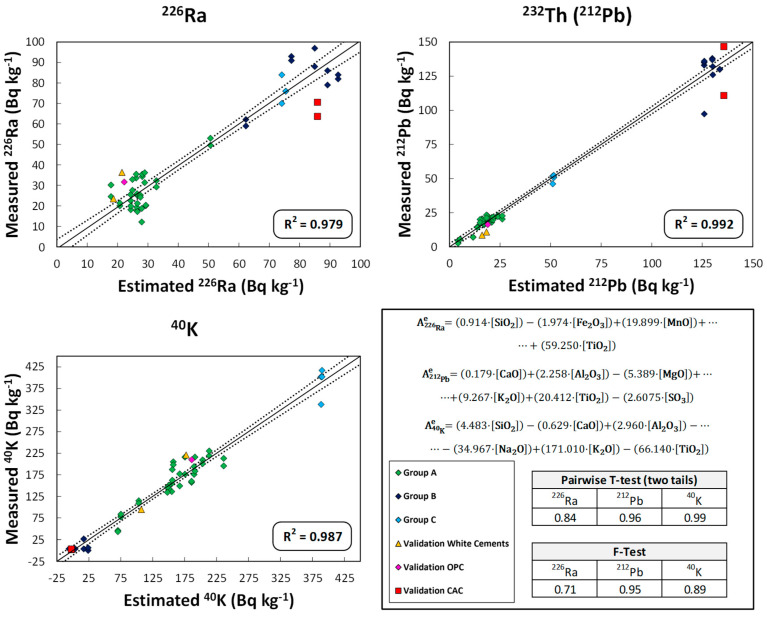
Graphical representation of the activity concentration values of ^226^Ra, ^232^Th(^212^Pb), and ^40^K measured experimentally versus those estimated using the models obtained that relate the chemical composition of the cements to the activity concentration of the different radionuclides. The models explain more than 98% of the dispersion. The clusters depicted by the confidence ellipses in Figure 3 are shown in the same color at the represented points (dark blue for CAC, green for CEM I and white cements, and light blue for CEM I type cements with higher ^210^Pb content). The comparison between the experimental and estimated values showed no significant differences, as the *p*-value for the paired *t*-test and Fisher’s F-test were higher than the significance value α 0.05. The values obtained in the validation of the method are included to check their agreement with the proposed model. The chemical composition and activity concentrations of the samples analyzed in validation are given in Tables S8 and S9 of the Supplementary Information.

**Figure 7:**
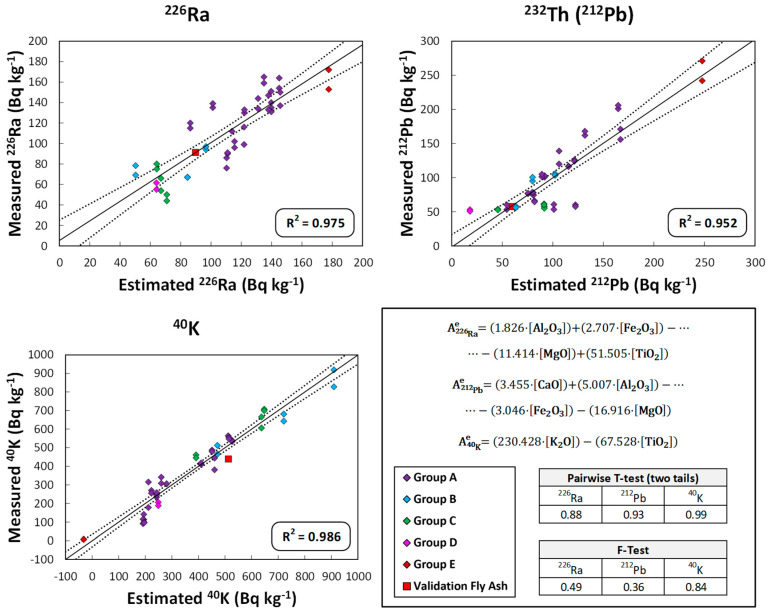
Graphical representation of the experimentally measured ^226^Ra, ^232^Th(^212^Pb), and ^40^K activity concentration values versus those estimated by the obtained models relating the chemical composition of the ashes to the activity concentration of the different radionuclides. The models explain more than 95% of the dispersion. The clusters represented by the confidence ellipses in the HJ-Biplot in Figure 4 are shown in the same color as the plotted points. The comparison between experimental and estimated values showed no significant differences, as the *p*-values for the paired *t*-test and Fisher’s F-test were higher than the α significance value of 0.05. The results obtained in the validation of the method are included to check their agreement with the proposed model.

**Figure 8:**
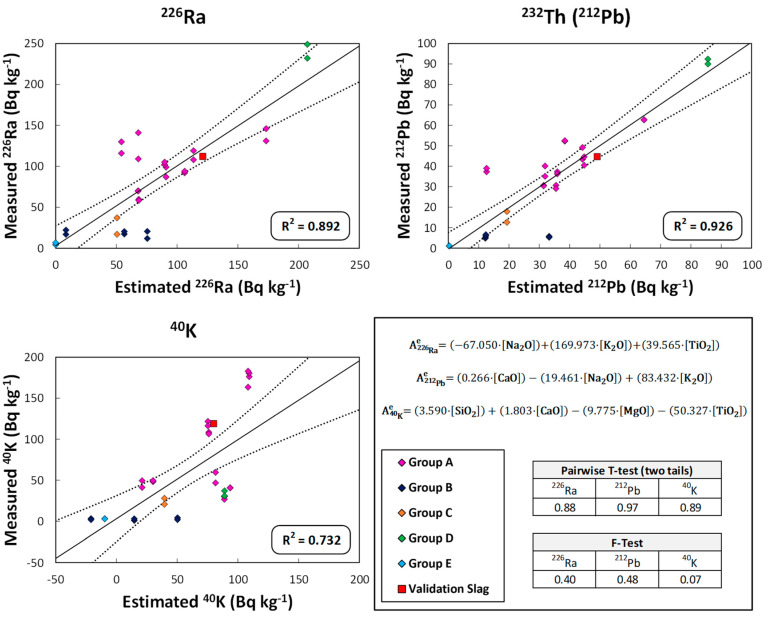
Graphical representation of the activity concentration values of ^226^Ra, ^232^Th(^212^Pb), and ^40^K measured experimentally versus those estimated using the models obtained that relate the chemical composition of the cements to the activity concentration of the different radionuclides. The models explain more than 73% of the dispersion. The clusters represented by the confidence ellipses in Figure 5 are shown with the same color at the represented points. Comparison between experimental and estimated values showed no significant differences, as the *p*-value for the paired *t*-test and Fisher’s F-test were above the α significance value of 0.05. The results obtained in the validation of the method are included for agreement with the proposed model.

Figure 6, Figure 7 and Figure 8 show the graphical representation of the activity concentration values of ^226^Ra, ^232^Th(^212^Pb), and ^40^K when measured experimentally versus those estimated using the models that relate the activity concentration of these radionuclides to the chemical composition in the cement, FA, and slag samples used in this study. The R^2^ values for each representation are also presented. The values represented in each figure are colored according to the groupings obtained in Figure 3, Figure 4 and Figure 5, respectively. The results obtained in the statistical tests used to evaluate the accuracy (Student’s *t*-test of paired results) and precision (Fisher’s F-test) of the set of values, based on the *p*-values for a significance level α of 0.05), are also presented.

The models obtained for the cements indicated that they accounted for 98% of the variability of the data represented, according to the values of the coefficients of determination R^2^ obtained in the multiple linear fit (Figure 6). The cements are represented according to the colors used in Figure 3, while validation of the white cement, grey OPC, and CAC is represented by a yellow triangle, a pink rhombus and a red square, respectively.

The model obtained for ^226^Ra for the cement samples takes into account the variables corresponding to the percentage of SiO_2_, Fe_2_O_3_, MnO, and TiO_2_. The remaining variables did not reach the required level of significance when applying the multiple regression model with backward stepwise regression. The inflation indices of the parameters obtained were higher than 10 in the case of Fe_2_O_3_ and TiO_2_ which, being correlated with each other (Figure 3), would present collinearity, as these variables are not independent (a more extensive explanation of the collinearity is presented in Annex 2 of the Supplementary Information). However, the reduction of variables in the backward stepwise multiple linear regression (with a significance value higher than 0.05) and the different chemical behavior between Fe_2_O_3_ and TiO_2_ were important criteria for keeping these variables in the model. In the case of ^232^Th(^212^Pb), the parameters that were not suppressed by the multiple linear models were CaO, Al_2_O_3_, MgO, TiO_2_, and SO_3_. Al_2_O_3_ and TiO_2_ obtained inflation indices well above 10, so they again showed collinearity. Finally, the model obtained for ^40^K took into account the variables corresponding to the concentrations of SiO_2_, CaO, Al_2_O_3_, Na_2_O, K_2_O, and TiO_2_. In this case, collinearity was found between Al_2_O_3_, TiO_2_, SiO_2_, CaO and K_2_O. However, these variables were correlated with each other according to factors (vectors) found in the HJ-Biplot (Figure 3), so collinearity would not affect the model.

As can be seen in Figure 7, the models obtained for the ashes explained 95% of the variability of the values represented by the R^2^ values obtained and again, with the colors used in the corresponding Figure 4. The backward stepwise multiple regression for ^226^Ra, ^232^Th(^212^Pb), and ^40^K eliminated more variables than in the case of the cements. However, the inflation indices obtained for the different variables in the three models (^226^Ra, ^232^Th(^212^Pb), and ^40^K) indicated an absence of collinearity between them. The paired *t*-tests and Fisher’s F-tests showed that there were no significant differences between the means and variances of the experimental values and those estimated by the proposed models. Validation with the new FA is represented by a red square.

The models, which relate the chemical composition of the slag samples to the activity concentration of ^226^Ra, ^232^Th(^212^Pb), and ^40^K, account for more than 73% of the variability of the values represented, according to the R^2^ values obtained (Figure 8) and with the colors corresponding to the colors in Figure 5. The coefficients of determination are lower than those obtained for cements and FA. The multiple linear backward stepwise fit again eliminated more variables than in the case of cement, and again, the variables did not suffer from collinearity between them. The experimental values and those predicted by the models obtained showed no significant differences, as the *p*-values obtained in the paired *t*-test and Fisher’s F-test were higher than the α significance level of 0.05. Again, validation is shown as a red square.

## 4. Discussion

The results obtained in our study have allowed us to verify the relationship between the chemical composition and the activity concentration of the natural radionuclides belonging to the natural radioactive series of uranium and thorium together with ^40^K in the cement, FA and slag samples studied. This has made it possible to establish specific models for each type of material (C, FA, and S) and each radionuclide (^226^Ra, ^212^Pb, and ^40^K) (Table 1).

The raw materials used in the manufacture of OPC cement clinker [66] are mainly limestone and clays [67]. The limestone, which mainly provides CaO, is made up of 95% calcite (or CaCO_3_), with MgCO_3_ as a secondary component, and may contain other mineralogical phases, such as quartz and muscovite [68,69]. The average limestone activity concentrations found in the literature ranged from 11.9 to 29.0 Bq kg^−1^ for ^226^Ra; from 1.6 to 20.2 Bq kg^−1^ for ^232^Th; and from 13.5 to 113.2 Bq kg^−1^ for ^40^K [70,71], with higher values found only in areas in India and Bangladesh, which presented average values of 67 Bq kg^−1^ for ^226^Ra, 60.8 Bq kg^−1^ for ^232^Th, and 496 Bq kg^−1^ for ^40^K. These values are consistent with the correlation found in Figure 3, in which the cements with higher CaO contents had lower levels of natural radioactive series activity concentration than those with lower CaO contents.

CACs are produced from limestone and bauxite. The correlation found between the radionuclides of the natural radioactive series and the Fe_2_O_3_, Al_2_O_3_, and TiO_2_ in these cements is more related to the bauxite used as raw material for this type of cement, whose chemical and radiological content depends on the areas where it is formed [72,73,74]. The mineralogical phases of bauxite are gibbsite (Al(OH)_3_), boehmite (γ-AlO(OH)), diaspore (α-AlOOH), hematite (Fe_2_O_3_), goethite (FeO(OH)), quartz (SiO_2_), rutile (TiO_2_), and kaolin (Al_2_Si_2_O_5_(OH)_4_) [75], which would imply a higher content of Fe_2_O_3_, Al_2_O_3_, and TiO_2_. The increase in the natural radionuclide content, with respect to OPC-type cements, would therefore be related to the presence of bauxite in the manufacturing process. Bauxites have a high content of radionuclides belonging to the radioactive thorium and uranium series, but not as high as ^40^K, which is consistent with the grouping shown in the HJ–Biplot (Figure 3) [76]. The ^40^K exhibited the expected behavior as it correlated with K_2_O and Na_2_O. With respect to the models obtained, it was observed that the oxides with the greatest weight in the ^226^Ra model were TiO_2_ and Fe_2_O_3_. TiO_2_ and Al_2_O_3_ were also the most important oxides in the ^212^Pb activity concentration model. Finally, K_2_O, Na_2_O, and TiO_2_ had the most weight on the ^40^K activity concentration according to the model obtained. The parameters obtained in the models would be consistent with the adsorption of ^226^Ra in the presence of Fe_2_O_3_ [74] and of ^232^Th in the presence of Al_2_O_3_ [77].

FAs are mainly composed of SiO_2_, Al_2_O_3_, and Fe_2_O_3_, with CaO content below 10% in ASTM F-type ashes [30], which are the ones mainly used in this study. Likewise, the percentages of MgO and TiO_2_ are very low in this type of ash [78]. The content in the naturally occurring radioactive uranium and thorium series would be related to the coal used in the power plant [4]. FA resulting from the burning of coals containing phosphate minerals, such as monazite or apatite, have a higher content of the thorium series, while those containing organic matter, such as lignite, predominantly contain the uranium series [31,39]. This fact, in addition to the composition of the coal itself, is due to the by-products generated during the burning process. The U associated with the organic matter would form volatile compounds such as UO_3_ [79]. Ra would also form volatile species such as Ra(OH)_2_ [80]. Therefore, both U and Ra would be concentrated in the finer particles of the FAs by condensation. Th would be bound to particles composed of zircon (ZrSiO_4_) [43] and adsorbed by the formation of ThCl_4_ due to the presence of organic material [81]. The ^40^K would be related to the presence of inorganic matter and, therefore, increasing U would decrease the ^40^K while the opposite would happen with Th [37]. The scores obtained for the FA analyzed in the HJ–Biplot (Figure 4) showed a high heterogeneity, reflecting the high clustering of points at the origin of the coordinates. This behavior would be consistent with the variability of coal burned in electrothermal power plants. The uranium and thorium series were correlated with each other along with the TiO_2_ and Al_2_O_3_ oxides, with no correlation observed with the other major oxides (Fe_2_O_3_, CaO, and SiO_2_). The correlation found between ^40^K, K_2_O, and SiO_2_ would be related to the inorganic matter in the starting coal. Our results did not show the relationship between SO_3_ and radioactive content that was observed by Kovacs et al. [42], since SO_3_ is perpendicular to the vectors of the natural radioactive series, which would show an absence of correlation.

FA18 was the FA with the highest activity concentration of the uranium and thorium series radionuclides and therefore scored separately from the rest. The models for both ^226^Ra and ^212^Pb activity concentration obtained weight for the majority of oxides in the FA together with TiO_2_. The oxides with the greatest weight in the ^40^K activity concentration model were K_2_O and TiO_2_.

Granulated blast-furnace slag (GBFS) is obtained from iron ore, which may contain clayey material, limestones and dolomites (used as melting fluxes), and the sulfur ash from coke, used as blast-furnace feed [44,46]. The radioactive content of this type of material has been little studied and varies between 16.1 and 167 Bq kg^−1^ for ^226^Ra, between 5.0 and 66.5 Bq kg^−1^ for ^232^Th, and between 96.1 and 235.6 Bq kg^−1^ for ^40^K [50,82,83]. The scores obtained in the HJ–Biplot (Figure 5) showed, like the FA, high variability and were again close to the origin of the coordinates, showing little influence of the factors found by the PCA. The uranium and thorium radioactive series together with ^40^K was correlated with decreasing Al_2_O_3_ and K_2_O percentages and an increasing MgO percentage. CaO and SiO_2_ showed no correlation with the radioactive content. The model obtained for ^226^Ra and ^212^Pb obtained the Na_2_O and K_2_O percentages as the most important factors, which is consistent with the correlations found between the variables in the HJ–Biplot (Figure 5). TiO_2_ again obtained a significant weight in the ^226^Ra model. The weights obtained in the ^40^K model would be those corresponding to the variables with which no correlation was found, i.e., SiO_2_, CaO, MgO, and TiO_2_. The percentage of ^40^K in this case would have no weight in the model.

S2 and S3 are steel slags whose chemical composition depends fundamentally on the starting material and the processes used to manufacture common and stainless steel. These slags usually contain oxides in different proportions, depending on their origin, with values of CaO (22–60%), SiO_2_ (11–37%), Fe_2_O_3_ (5–38%), MgO (4–12%), MnO (2–5%), and P_2_O_5_ (0.5–2%) [84,85]. These slags contained traces of ^241^Am, which cannot be from a natural source and could therefore be from accidental contamination, which is usually due to abandoned neutron sources (^241^Am/Be), although this hypothesis cannot be supported experimentally [86].

**Figure 9:**
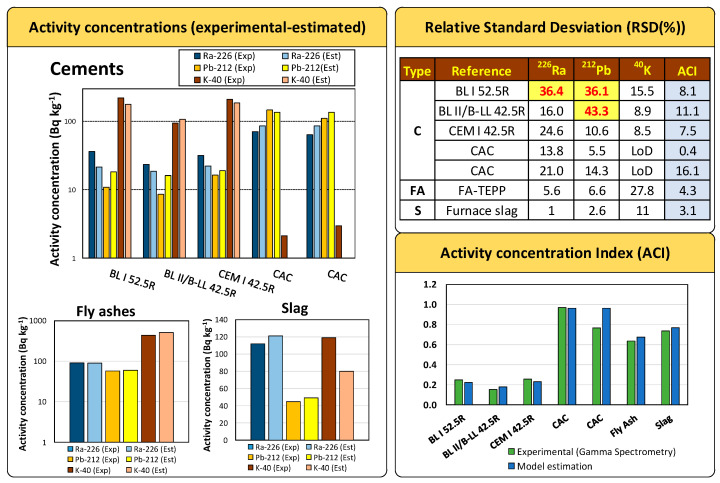
Validation of activity concentrations for ^226^Ra, ^212^Pb, and ^40^K, and ACI experimentally determined by gamma spectrometry and those estimated by the proposed models. The validation criterion for satisfactory results was an RSD(%) value of less than or equal to 30%. Eighty-nine percent of the concentrations met the acceptance criterion. The ^40^K was also accepted for the 2 samples with an activity concentration value below the limit of detection (LoD). The RSD(%) values obtained for the ACIs were all satisfactory at less than 16.1%.

For the validation of the proposed models, five cements, one FA and one slag—whose estimated ^226^Ra, ^232^Th(^212^Pb), and ^40^K activity concentration values and ACI were calculated from the proposed models (Table 1)—were used as mentioned above. The activity concentrations of ^226^Ra, ^212^Pb and ^40^K were within the range of values used in the model, as extrapolations in this type of model are not correct. These values were plotted in Figure 6, Figure 7 and Figure 8 and are presented in more detail in Figure 9.

The RSD(%) values obtained for the experimental ^226^Ra, ^212^Pb, and ^40^K activity concentrations and those estimated from the models established for each material and each radionuclide met the 30% criterion set in Section 2.5. Eighty-nine percent of the RSD(%) values obtained for the experimental ^226^Ra, ^212^Pb, and ^40^K activity concentrations and those estimated from the models established for each material and each radionuclide met the 30% criterion set in Section 2.5.

These results reflect a very satisfactory estimation of activity concentration values for the three matrices studied. The ^40^K concentration in the CACs could not be validated as they were below the LoD. However, the model predicted negative values close to 0, which are therefore consistent with the results obtained. The RSD(%) values obtained for the samples used in the validation of the determined models were higher than 30% for ^226^Ra and ^232^Th(^212^Pb) in the case of BL I 52.5R cement and for ^226^Ra in the case of BL-II/B-LL 42.5R cement. The ^226^Ra activity concentration estimated by the model (21.44 Bq kg^−1^) in BL I 52.5R cement was lower than that obtained experimentally (36.30 ± 4.6 Bq kg^−1^). This discrepancy may be due to the greater uncertainty in the model based on the dispersion of the values used (Figure 6). The differences were nevertheless close to the 30% criterion. On the other hand, the estimated activity concentrations for ^212^Pb were 18.26 Bq kg^−1^ and 16.08 Bq kg^−1^ versus the experimental values of 10.84 ± 0.47 Bq kg^−1^ and 8.54 ± 0.35 Bq kg^−1^ for the BL I 52.5R and BL-II/B-LL 42.5R cements, respectively. The discrepancy could be due to the low activity concentration of these samples with respect to the set of results grouped at the activity concentration of 20 Bq kg^−1^ (Figure 6). However, despite the aforementioned discrepancies, the authors consider the model estimates to be satisfactory and could improve in accuracy if the number of samples used by the model were increased.

The ACIs estimated for the six materials tested were satisfactory as they all obtained an RSD(%) below 16.1%, which confirms the validity of the model established for this parameter, which was our initial hypothesis.

This paper, therefore, establishes the basis for predicting the ACI of the cements, ashes, and slags via the chemical composition of these materials and the proposed models. This would allow us to make decisions regarding the ACI of a given material based on its chemical composition and subsequently confirm it with gamma spectrometry, which involves more analysis time and is not as common a technique as XRF.

## 5. Conclusions

The study found a relationship between chemical composition and radioactivity (specifically, the activity concentration index-ACI) in anhydrous cements, coal combustion FA, and slags of different origins (steel and steelworks). The correlations found are consistent with the interaction between the radionuclides of the uranium and thorium radioactive series, as well as ^40^K, and the oxides that define the chemical composition of the samples and their starting materials.

In the case of cements, it was found that as the concentration of Fe_2_O_3_, TiO_2_, and Al_2_O_3_ increased, the activity concentrations of radionuclides belonging to the uranium and thorium series also increased. However, these activity concentrations decreased as the concentration of CaO and SO_3_ increased. In the case of ^40^K, a correlation was found with SiO_2_, MnO, MgO, and K_2_O.

In the case of FAs, the correlations between the radionuclides of the natural radioactive series increased as the concentration of TiO_2_ and Al_2_O_3_ increased, and in the case of ^40^K with K_2_O.

Finally, in the case of slags, it was observed that all the studied natural radionuclides increased as the concentration of Al_2_O_3_, K_2_O, and Na_2_O increased.

The models proposed in this study provide a new means of determining the suitability of material from a radiological protection perspective. They also offer the ability to validate the activity concentrations of ^226^Ra, ^212^Pb, and ^40^K obtained via gamma spectrometry, within the range of values considered in this study, which are necessary for the final calculation of the ACI. The models were validated using five cements, one FA and one slag with relative percentage deviations (RSD(%)) equal to or less than 30% for 89% of the activity concentrations and 100% of the ACI determined.

This study provides the basis for a new approach utilizing mathematical models based on neural networks, similar to the ones proposed in this study, but less restrictive than traditional statistical models. This approach has the potential to improve ACI prediction, taking into account the radiological protection perspective, and is a subject worthy of further research, as chemical analysis is a cost-effective, readily available, and less time-consuming alternative to radioactive analysis.

## Figures and Tables

**Table 1:** Models established to determine the activity concentration of ^226^Ra, ^212^Pb, and ^40^K in C, FA, and S from their chemical composition.

Material	Model	Radionucleid
C	ARa226e=0.914·SiO2−1.974·Fe2O3+19.899·MnO+59.250·TiO2	^226^Ra
	APb212e=0.179·CaO+2.258·Al2O3−5.389·MgO+… $\ldots +\left(9.267\cdot \left[K_{2}O\right]\right)+\left(20.412\cdot \left[{\mathrm{TiO}}_{2}\right]\right)-\left(2.6075\cdot \left[{\mathrm{SO}}_{3}\right]\right)$	^212^Pb
	AK40e=4.483·SiO2−0.629·CaO+2.960·Al2O3−… $\ldots -\left(34.967\cdot \left[{\mathrm{Na}}_{2}O\right]\right)+\left(171.010\cdot \left[K_{2}O\right]\right)-\left(66.140\cdot \left[{\mathrm{TiO}}_{2}\right]\right)$	^40^K
FA	ARa226e=1.826·Al2O3+2.707·Fe2O3−11.414·MgO+51.505·TiO2	^226^Ra
	APb212e=3.455·CaO+5.007·Al2O3−3.046·Fe2O3−16.916·MgO	^212^Pb
	AK40e=230.428·K2O−67.528·TiO2	^40^K
S	ARa226e=−67.050·Na2O+169.973·K2O+39.565·TiO2	^226^Ra
	APb212e=0.266·CaO−19.461·Na2O+83.432·K2O	^212^Pb
	AK40e=3.590·SiO2+1.803·CaO−9.775·MgO−50.327·TiO2	^40^K

## Data Availability

The data presented in this study and Supplementary Information are available on request from the corresponding author.

## Supplementary Materials

The following supporting information can be downloaded at: https://www.mdpi.com/article/10.3390/ma16072677/s1, Table S1: Type and chemical composition (wt. %) of the analysed Cements, Table S2: Type and chemical composition (wt. %) of the analysed Fly Ashes, Table S3: Type and chemical composition (wt. %) of the analysed Slags, Table S4: Activity concentration for the gamma emitters in the naturally occurring ^238^U, ^235^U, ^232^Th and ^40^K series in cements, Table S5: Activity concentration for the gamma emitters in the naturally occurring ^238^U, ^235^U, ^232^Th and ^40^K series in Fly Ashes, Table S6: Activity concentration for the gamma emitters in the naturally occurring ^238^U, ^235^U, ^232^Th and ^40^K series in Slags, Table S7: Activity concentration for the gamma emitters in the naturally occurring ^238^U, ^235^U, ^232^Th and ^40^K series, including ^241^Am, in Slags, Table S8: Chemical composition (wt. %) of the analysed Cements, Fly Ashes and Slags used for the models validations, Table S9: Experimental Activity concentration for the gamma emitters in the naturally occurring ^238^U, ^235^U, ^232^Th and ^40^K series, in Cements, Fly Ashes and Slags used for the models validations; Table S10: Correlation matrix between independent variables (wt. %) for Cements, Table S11: Correlation matrix between independent variables (wt. %) for Fly Ashes, Table S12: Correlation matrix between independent variables (wt. %) for Slags and Table S13: Calculated VIF for the variables in the ^226^Ra, ^232^Th (^212^Pb) and ^40^K models for cements, FA and S. Reference [87] are cited in the supplementary materials.
