# Supplementary material for: New Approach to Determine the Activity Concentration Index in Cements, Fly Ashes, and Slags on the Basis of Their Chemical Composition
Source: Materials (Basel). 2023 Mar 28;16(7):2677. doi: 10.3390/ma16072677 (PMC10095990; doi:10.3390/ma16072677)
Supplement: Supplementary file 1 [file materials-16-02677-s001.zip › materials-2274634-supplementary.pdf]

Annex 1

Table S1. Type and chemical composition (wt. %) of the analysed Cements.

	Type	SiO_2	CaO	Al_2O_3	Fe_2O_3	MgO	MnO	Na_2O	K_2O	TiO_2	SO_3	Other Oxides	LoI*
**C17**	Belitic cement 30%FA	37.46	28.56	14.31	5.82	2.00	0.05	1.29	1.74	0.85	3.47	0.36	3.31
**C18**	CEM I 52.5 R	20.00	64.08	4.37	2.53	1.87	0.09	0.13	0.77	0.21	2.89	0.45	2.78
**C19**	CEM I	37.93	28.40	14.48	5.77	1.99	0.06	1.31	1.74	0.86	3.52	0.36	3.30
**C20**	CEM I 42.5R	19.97	56.74	6.16	3.21	1.21	0.07	0.33	0.86	0.25	3.75	0.42	6.08
**C21**	CEM I 52.5 R	19.63	64.37	5.66	2.43	0.86	0.06	0.04	0.64	0.23	3.59	0.23	3.05
**C22**	CAC	4.53	37.34	38.88	12.46	0.63	-	0.03	0.13	1.65	-	0.24	0.25
**C23**	CEM I 42.5 R	19.47	63.83	5.73	2.68	0.90	0.05	0.05	0.63	0.22	3.15	0.17	3.57
**C24**	CEM I 52-5 N-SR	20.55	63.66	3.84	4.20	0.75	0.16	0.01	0.58	0.18	3.06	0.19	3.32
**C25**	CEM I 42.5 R	19.84	63.78	5.38	2.43	1.48	0.07	0.04	0.70	0.24	3.33	0.26	2.78
**C26**	CEM I 52.5R R-SR	20.59	64.41	3.72	3.23	1.50	-	0.10	0.60	0.21	3.03	0.43	3.01
**C27**	CAC	4.40	39.43	37.9	14.93	0.76	0.19	-	0.09	1.80	0.02	0.32	0.68

**LoI: Lost on Ignition*

Table S2. Type and chemical composition (wt. %) of the analysed Fly Ashes.

	Type*	SiO_2	CaO	Al_2O_3	Fe_2O_3	MgO	MnO	Na_2O	K_2O	TiO_2	SO_3	Other Oxides	LoI**
**FA12**	F	51.80	2.69	25.30	13.43	0.91	0.04	0.45	2.46	0.81	-	0.07	1.72
**FA13**	F	53.90	5.32	26.00	6.01	1.73	-	0.36	2.38	1.30	0.45	0.69	1.68
**FA14**	F	56.97	9.67	18.80	4.10	1.39	0.12	1.66	3.00	0.80	0.45	1.14	1.93
**FA15**	F	55.96	10.55	18.60	4.25	1.50	0.12	1.40	3.03	0.75	0.47	1.05	2.31
**FA16**	F	45.58	9.17	32.25	4.39	0.76	0.10	0.98	1.17	1.16	0.70	0.44	3.30
**FA17**	F	44.62	8.24	33.05	4.95	0.84	0.10	0.96	1.23	1.30	0.64	0.66	3.42
**FA18**	F	42.71	2.21	48.86	1.49	-	-	-	0.34	1.64	0.14	0.37	2.25
**FA19**	F	54.49	1.37	29.55	5.80	1.15	-	-	2.75	1.58	-	0.47	2.84
**FA20**	F	56.12	1.03	29.29	5.10	0.79	-	-	2.42	1.58	-	0.46	2.61
**FA21**	F	41.55	6.37	22.97	23.26	0.70	0.06	0.36	1.33	0.95	0.75	0.27	1.42
**FA22**	Landfill FA	23.95	21.84	10.08	15.41	6.60	0.18	0.36	0.58	0.76	1.56	0.78	17.88
**FA23**	F	59.04	2.01	22.50	4.32	0.87	0.02	0.85	1.30	0.84	0.17	0.09	2.32
**FA24**	F	52.77	6.51	18.10	7.14	2.71	0.06	1.13	1.94	0.83	0.47	0.41	7.93
**FA25**	F	51.47	2.38	25.61	14.42	1.01	0.04	-	2.47	0.80	-	0.23	1.57
**FA26**	F	51.94	4.37	25.05	6.38	1.89	0.06	1.24	2.12	1.16	0.11	1.69	3.87

**According to ASTM C618 **LoI: Lost on Ignition*

Table S3. Type and chemical composition (wt. %) of the analysed Slags.

	Type	SiO_2	CaO	Al_2O_3	Fe_2O_3	MgO	MnO	Na_2O	K_2O	TiO_2	SO_3	ZnO	Other Oxides	LoI*
**S6**	Blast furnace Vitreous Slag	32.32	45.70	9.59	0.54	7.13	0.17	0.32	0.46	0.94	1.59	-	0.31	0.95
**S7**	Blast furnace Vitreous Slag	24.05	28.42	9.97	1.60	1.38	1.41	-	0.29	0.29	1.53	0.01	0.28	30.78
**S8**	Blast furnace Vitreous Slag	34.36	34.13	9.97	2.19	5.65	0.16	1.54	0.79	0.42	2.24	-	0.21	8.35
**S9**	Nickel Slag	52.92	0.98	2.98	20.39	20.60	0.44	-	-	-	0.10	0.02	1.38	0.19
**S10**	Blast furnace Vitreous Slag	36.15	18.22	15.10	18.67	3.69	0.70	0.61	1.11	0.75	0.53	1.29	1.95	2.57
**S11**	Blast furnace Vitreous Slag	16.90	46.39	5.55	2.06	15.80	0.52	-	-	0.22	1.77	0.02	0.17	10.58
**S12**	Blast furnace Vitreous Slag	33.79	38.78	10.30	0.63	10.50	0.94	-	0.31	1.34	3.08	-	0.12	0.70
**S13**	Blast furnace Vitreous Slag	34.52	42.21	10.50	0.67	8.30	0.17	-	0.29	0.85	1.74	-	0.13	0.60
**S14**	Blast furnace Vitreous Slag	38.23	35.30	12.00	0.79	7.23	1.41	-	0.66	1.09	0.80	-	0.16	2.29
**S15**	Blast furnace Vitreous Slag	34.96	41.12	10.50	0.45	8.90	0.16	-	0.25	0.47	1.28	-	0.10	1.75
**S16**	Blast furnace Vitreous Slag	34.95	42.47	12.00	0.49	7.73	0.17	-	0.33	0.65	0.86	-	0.15	0.17
**S17**	Steel slag	12.70	39.06	16.10	18.52	3.99	3.71	-	0.11	0.75	0.29	-	1.16	3.59

**LoI: Lost on Ignition*

Table S4. Activity concentration for the gamma emitters in the naturally occurring ^{238}U, ^{235}U, ^{232}Th and ^{40}K series in cements.

**Series**		**^{238}U Radiactive Serie**					**^{235}U**	**^{232}Th Radiactive Serie**			**^{40}K**
**Cements**		**^{234}Th**	**^{226}Ra**	**^{214}Pb**	**^{214}Bi**	**^{210}Pb**		**^{228}Ac**	**^{212}Pb**	**^{208}Tl**	
**C17**	A	79.2 ± 9.3	70 ± 10	72.0 ± 5.5	64.8 ± 2.1	83 ± 13	< 3.5	42.4 ± 1.6	46.1 ± 3.7	18.6 ± 1.2	338 ± 15
	B	74.5 ± 9.0	84 ± 11	81.3 ± 6.3	75.5 ± 2.8	85 ± 13	< 3.0	49.4 ± 1.7	51.8 ± 4.3	20.8 ± 1.4	402 ± 19
**C18**	A	32.2 ± 5.7	25.2 ± 7.8	27.5 ± 2.2	26.3 ± 1.2	28.9 ± 6.9	< 4.7	20.0 ± 1.0	20.0 ± 1.7	8.51 ± 0.64	216 ± 11
	B	26.0 ± 4.1	24.2 ± 4.9	23.6 ± 1.8	22.27 ± 0.85	24.3 ± 4.9	< 2.1	16.03 ± 0.70	17.7 ± 1.4	7.55 ± 0.50	176.3 ± 8.2
**C19**	A	81.1 ± 9.5	76 ± 11	79.6 ± 6.2	73.3 ± 2.9	89 ± 14	3.98 ± 0.51	48.4 ± 2.1	50.7 ± 4.2	20.9 ± 1.4	402 ± 19
	B	81 ± 10	76 ± 12	81.6 ± 6.3	76.1 ± 2.7	87 ± 14	< 6.1	50.0 ± 2.0	52.3 ± 4.3	20.9 ± 1.4	417 ± 19
**C20**	A	41.7 ± 5.8	34.3 ± 6.2	32.7 ± 2.5	30.2 ± 1.1	32.7 ± 5.9	< 2.5	16.27 ± 0.71	17.9 ± 1.5	7.81 ± 0.52	183.2 ± 8.5
	B	43.5 ± 6.4	35.4 ± 7.0	36.5 ± 2.8	32.3 ± 1.7	37.4 ± 6.9	< 3.2	19.1 ± 1.0	19.6 ± 1.7	8.06 ± 0.69	216 ± 11
**C21**	A	28.7 ± 6.0	< 17.1	18.1 ± 1.6	15.6 ± 1.0	28.4 ± 7.7	< 5.7	19.4 ± 1.2	21.5 ± 1.8	9.02 ± 0.73	205 ± 11
	B	21.3 ± 4.8	18.7 ± 5.7	15.3 ± 1.2	13.02 ± 0.69	18.1 ± 4.9	< 2.8	18.29 ± 0.86	19.8 ± 1.6	7.72 ± 0.53	198 ± 10
**C22**	A	70.3 ± 8.3	93 ± 11	87.0 ± 6.6	82.6 ± 2.6	24.7 ± 5.4	< 3.9	128.3 ± 4.4	133 ± 11	55.8 ± 3.4	< 0.0
	B	79 ± 10	91 ± 13	90.7 ± 6.9	85.3 ± 2.7	29.3 ± 7.2	< 3.8	130.9 ± 4.4	135 ± 11	56.2 ± 3.5	< 8.9
**C23**	A	18.4 ± 2.7	17.2 ± 3.4	17.3 ± 1.3	14.15 ± 0.55	10.4 ± 2.6	< 1.6	18.28 ± 0.70	19.9 ± 1.6	7.93 ± 0.50	162.2 ± 7.3
	B	19.4 ± 3.7	21.2 ± 4.7	17.0 ± 1.3	15.03 ± 0.77	< 9.2	< 2.6	21.6 ± 1.4	21.8 ± 1.8	9.41 ± 0.74	187.0 ± 9.5
**C24**	A	19.2 ± 3.9	18.1 ± 7.3	17.2 ± 1.4	14.67 ± 0.92	17.4 ± 5.3	< 4.5	16.05 ± 0.91	16.1 ± 1.3	5.88 ± 0.52	142.9 ± 7.7
	B	24.0 ± 4.1	19.9 ± 5.0	16.6 ± 1.5	16.18 ± 0.89	23.3 ± 5.3	< 2.4	16.1 ± 1.1	16.6 ± 1.4	7.26 ± 0.58	150.8 ± 7.8
**C25**	A	31.1 ± 4.8	36.2 ± 5.9	31.9 ± 2.6	30.2 ± 1.4	26.7 ± 5.6	< 2.7	22.0 ± 1.3	23.3 ± 2.0	8.52 ± 0.72	177.0 ± 9.1
	B	31.7 ± 4.9	31.4 ± 6.0	26.0 ± 2.0	23.76 ± 0.92	23.6 ± 4.9	< 2.5	19.08 ± 0.81	21.2 ± 1.7	8.23 ± 0.54	149.1 ± 7.2
**C26**	A	35.2 ± 4.8	27.6 ± 5.3	30.2 ± 2.4	26.5 ± 1.3	34.5 ± 6.4	< 2.4	14.8 ± 1.5	14.5 ± 1.2	5.62 ± 0.47	146.1 ± 7.5
	B	35.6 ± 5.6	32.9 ± 7.7	28.2 ± 2.2	26.8 ± 1.2	28.7 ± 6.5	< 4.1	11.53 ± 0.71	14.1 ± 1.2	5.28 ± 0.44	134.4 ± 7.0
**C27**	A	90 ± 11	88 ± 12	89.0 ± 6.8	82.4 ± 2.8	62 ± 11	< 4.1	128.0 ± 4.6	136 ± 11	50.1 ± 3.1	< 5.4
	B	85 ± 11	97 ± 14	91.2 ± 7.0	87.5 ± 2.9	59 ± 11	< 5.3	131.0 ± 4.5	97.3 ± 7.9	50.3 ± 3.1	< 0.0

Uncertainties are expressed for a coverage factor of k=2.

Table S5. Activity concentration for the gamma emitters in the naturally occurring ^{238}U, ^{235}U, ^{232}Th and ^{40}K series in Fly Ashes.

**Series**		**^{238}U Radiactive Serie**					**^{235}U**	**^{232}Th Radiactive Serie**			**^{40}K**
**Fly Ashes**		**^{234}Th**	**^{226}Ra**	**^{214}Pb**	**^{214}Bi**	**^{210}Pb**		**^{228}Ac**	**^{212}Pb**	**^{208}Tl**	
**FA12**	A	120 ± 13	112 ± 14	115.0 ± 8.8	106.5 ± 3.5	121 ± 18	5.2 ± 1.0	76.1 ± 2.9	78.4 ± 6.4	29.1 ± 1.9	564 ± 25
**FA13**	A	80.0 ± 9.2	90 ± 11	81.9 ± 6.2	76.3 ± 2.4	94 ± 14	< 3.0	51.8 ± 1.9	53.4 ± 4.3	20.2 ± 1.3	381 ± 17
	B	93 ± 10	91 ± 11	92.7 ± 7.1	86.5 ± 2.6	102 ± 15	< 2.8	58.7 ± 2.3	60.6 ± 5.0	22.9 ± 1.5	444 ± 20
**FA14**	A	58.8 ± 8.1	44 ± 10	56.4 ± 4.4	54.4 ± 2.0	54.5 ± 8.8	< 5.4	54.2 ± 2.1	57.5 ± 4.7	24.4 ± 1.6	665 ± 29
	B	54.1 ± 6.9	50.0 ± 8.0	55.8 ± 4.3	50.0 ± 1.6	52.6 ± 8.3	< 3.5	50.5 ± 1.8	55.1 ± 4.5	22.7 ± 1.4	606 ± 26
**FA15**	A	47.2 ± 7.3	66 ± 11	63.0 ± 4.9	59.7 ± 2.3	59 ± 10	< 6.1	59.1 ± 2.3	61.4 ± 5.0	25.8 ± 1.7	707 ± 31
	B	60.1 ± 8.0	53.9 ± 9.0	59.6 ± 4.7	56.4 ± 2.3	49.4 ± 8.2	< 3.8	58.1 ± 2.6	60.1 ± 4.9	25.3 ± 1.7	699 ± 31
**FA16**	A	146 ± 18	116 ± 20	136 ± 10	133.9 ± 4.5	142 ± 22	< 9.0	162.2 ± 5.7	171 ± 14	70.1 ± 4.4	115 ± 10
	B	141 ± 16	99 ± 16	122.5 ± 9.3	111.8 ± 3.5	120 ± 18	< 5.3	143.2 ± 4.9	156 ± 13	63.4 ± 3.9	91.3 ± 6.8
**FA17**	A	167 ± 18	144 ± 19	158 ± 12	148.0 ± 4.5	214 ± 31	6.0 ± 2.9	195.3 ± 6.8	201 ± 16	84.3 ± 5.2	99.2 ± 8.1
	B	162 ± 18	134 ± 20	164 ± 12	154.5 ± 4.7	210 ± 31	< 6.7	196.0 ± 6.6	206 ± 17	84.3 ± 5.2	95.7 ± 8.1
**FA18**	A	184 ± 20	153 ± 20	165 ± 13	147.2 ± 4.3	149 ± 22	6.74 ± 0.94	225.9 ± 7.5	242 ± 20	99.4 ± 6.1	< 11.4
	B	191 ± 21	172 ± 23	187 ± 14	175.4 ± 5.8	182 ± 27	< 6.4	259 ± 11	271 ± 22	113.7 ± 7.1	< 10.0
**FA19**	A	130 ± 16	147 ± 20	137 ± 10	129.0 ± 4.2	152 ± 23	< 7.5	109.2 ± 3.9	116.8 ± 9.5	49.5 ± 3.1	538 ± 25
	B	136 ± 15	133 ± 16	141 ± 11	131.5 ± 4.1	141 ± 21	5.39 ± 0.75	110.2 ± 4.1	116.2 ± 9.4	35.3 ± 2.2	533 ± 24
**FA20**	A	151 ± 17	140 ± 19	160 ± 12	155.3 ± 4.7	158 ± 23	< 4.5	120.3 ± 4.1	126 ± 10	54.1 ± 3.4	487 ± 22
	B	157 ± 17	151 ± 19	156 ± 12	145.4 ± 4.8	144 ± 21	6.7 ± 1.5	118.4 ± 4.5	124 ± 10	37.6 ± 2.4	479 ± 22
**FA21**	A	157 ± 17	137 ± 17	146 ± 11	135.6 ± 4.9	94 ± 14	5.60 ± 0.70	49.6 ± 1.7	53.1 ± 4.3	21.6 ± 1.3	252 ± 11
	B	167 ± 18	150 ± 19	161 ± 12	149.1 ± 4.7	106 ± 16	7.60 ± 0.92	56.4 ± 2.5	60.2 ± 4.9	24.8 ± 1.7	234 ± 12
**FA22**	A	37.2 ± 5.5	64.1 ± 8.6	50.1 ± 4.0	43.7 ± 1.7	51.7 ± 8.5	< 3.7	42.7 ± 1.9	43.9 ± 3.6	17.9 ± 1.3	89.9 ± 7.1
	B	42.7 ± 6.6	48.2 ± 8.5	46.5 ± 3.6	42.6 ± 1.9	50.2 ± 8.4	< 3.4	35.8 ± 1.4	38.9 ± 3.2	16.1 ± 1.0	111.6 ± 5.8
**FA23**	A	120 ± 13	115 ± 14	120.2 ± 9.2	110.8 ± 3.5	125 ± 18	5.3 ± 1.0	96.4 ± 3.5	100.6 ± 8.2	43.8 ± 2.8	245 ± 12
	B	123 ± 14	120 ± 16	120.6 ± 9.2	114.6 ± 3.6	124 ± 19	< 5.6	97.9 ± 3.4	102.9 ± 8.3	44.0 ± 2.7	258 ± 13
**FA24**	A	76.8 ± 9.0	75 ± 10	71.7 ± 5.6	67.0 ± 2.6	81 ± 12	< 3.8	51.2 ± 2.4	53.7 ± 4.4	23.4 ± 1.6	461 ± 21
	B	69.6 ± 8.3	80 ± 10	70.9 ± 5.5	66.3 ± 2.4	75 ± 11	< 3.6	50.4 ± 2.1	52.7 ± 4.3	23.5 ± 1.6	445 ± 20
**FA25**	A	123 ± 15	96 ± 16	112.5 ± 8.6	107.2 ± 3.6	101 ± 16	< 6.6	71.4 ± 2.7	76.5 ± 6.2	34.0 ± 2.2	555 ± 25
	B	118 ± 13	102 ± 14	114.2 ± 8.8	103.7 ± 3.7	118 ± 18	4.8 ± 1.2	74.0 ± 2.9	77.1 ± 6.3	34.3 ± 2.3	547 ± 25
**FA26**	A	185 ± 20	135 ± 19	144 ± 11	134.1 ± 4.1	222 ± 32	6.31 ± 0.74	95.7 ± 3.5	101.2 ± 8.2	41.8 ± 2.6	409 ± 19
	B	177 ± 19	139 ± 19	151 ± 12	143.6 ± 4.3	232 ± 34	< 4.7	95.4 ± 3.3	105.0 ± 8.5	43.5 ± 2.7	418 ± 19

Uncertainties are expressed for a coverage factor of k=2.

Table S6. Activity concentration for the gamma emitters in the naturally occurring ^{238}U, ^{235}U, ^{232}Th and ^{40}K series in Slags.

Series		^{238}U Radiactive Serie					^{235}U	^{232}Th Radiactive Serie			^{40}K
**Slags**		^{234}Th	^{226}Ra	^{214}Pb	^{214}Bi	^{210}Pb		^{228}Ac	^{212}Pb	^{208}Tl	
**S6**	A	133 ± 15	92 ± 16	125 ± 10	117.9 ± 3.7	< 13.0	< 4.8	46.0 ± 1.8	49.2 ± 4.0	20.0 ± 1.3	59.6 ± 5.0
	B	121 ± 13	94 ± 13	108.9 ± 8.3	95.4 ± 2.9	< 12.2	5.2 ± 1.0	38.0 ± 1.4	43.4 ± 3.5	17.9 ± 1.1	46.8 ± 3.1
**S7**	A	70.8 ± 8.7	70 ± 10	67.2 ± 5.1	58.6 ± 2.0	23.3 ± 5.1	< 3.7	28.5 ± 1.2	30.6 ± 2.5	12.35 ± 0.76	176.3 ± 8.9
	B	68.1 ± 8.6	70 ± 11	66.0 ± 5.1	58.2 ± 2.1	23.8 ± 5.4	4.7 ± 1.3	27.5 ± 1.3	30.4 ± 2.5	11.79 ± 0.81	180.4 ± 9.3
**S8**	A	61.5 ± 8.0	59.6 ± 9.4	59.9 ± 4.7	55.1 ± 2.4	38.2 ± 6.9	< 4.1	42.0 ± 2.3	44.6 ± 3.6	18.1 ± 1.3	183 ± 11
	B	55.5 ± 7.4	58.3 ± 9.3	53.8 ± 4.1	48.0 ± 1.8	30.7 ± 5.8	< 3.6	38.2 ± 1.6	40.5 ± 3.3	16.7 ± 1.1	163.4 ± 8.5
**S9**	A	< 5.0	< 5.9	< 1.0	< 1.1	< 4.7	< 1.8	< 1.4	1.02 ± 0.11	0.378 ± 0.053	3.28 ± 0.65
	B	< 5.4	6.5 ± 2.6	< 1.2	< 1.0	< 5.1	< 1.9	< 1.5	1.09 ± 0.25	< 0.4	< 4.4
**S10**	A	292 ± 32	249 ± 34	283 ± 22	265.1 ± 7.9	137 ± 21	12.6 ± 5.1	85.8 ± 3.2	92.4 ± 7.5	38.6 ± 2.4	37.1 ± 6.5
	B	299 ± 32	232 ± 31	274 ± 21	257.4 ± 7.7	147 ± 22	8.2 ± 1.5	82.9 ± 3.5	90.0 ± 7.3	27.9 ± 1.8	30.8 ± 6.1
**S11**	A	28.0 ± 4.1	16.8 ± 4.5	16.7 ± 1.3	13.65 ± 0.62	< 6.7	< 2.0	4.81 ± 0.33	5.95 ± 0.50	2.33 ± 0.19	< 4.8
	B	28.5 ± 3.4	22.1 ± 3.7	19.4 ± 1.6	17.77 ± 0.86	< 4.5	< 1.6	5.89 ± 0.32	6.55 ± 0.59	2.73 ± 0.26	< 2.8
**S12**	A	107 ± 13	119 ± 16	116.4 ± 8.9	107.5 ± 3.5	52.6 ± 9.0	< 6.2	33.1 ± 1.4	37.3 ± 3.0	15.9 ± 1.1	41.3 ± 4.8
	B	114 ± 12	108 ± 13	114.4 ± 8.7	107.6 ± 3.3	50.0 ± 7.8	4.67 ± 0.57	34.1 ± 1.4	36.3 ± 3.0	11.09 ± 0.74	49.5 ± 3.8
**S13**	A	110 ± 12	99 ± 14	103.3 ± 7.9	95.3 ± 3.0	39.2 ± 7.3	< 3.7	29.1 ± 1.2	30.7 ± 2.5	13.76 ± 0.93	108.4 ± 6.0
	B	99 ± 11	87 ± 12	94.9 ± 7.3	86.9 ± 3.0	34.3 ± 6.5	3.85 ± 0.67	26.7 ± 1.6	29.1 ± 2.4	12.8 ± 1.0	106.4 ± 6.5
**S14**	A	138 ± 16	146 ± 19	146 ± 11	139.8 ± 4.5	< 17.3	< 7.2	59.0 ± 2.3	62.7 ± 5.1	27.5 ± 1.8	121.7 ± 8.0
	B	150 ± 17	131 ± 17	144 ± 11	133.7 ± 4.3	15.2 ± 4.9	< 3.0	58.5 ± 2.6	62.7 ± 5.1	27.6 ± 1.8	116.3 ± 7.6
**S15**	A	132 ± 15	141 ± 17	121.1 ± 9.2	115.9 ± 3.5	32.0 ± 6.5	< 3.9	38.7 ± 1.5	40.1 ± 3.3	15.6 ± 1.0	30.2 ± 3.5
	B	129 ± 14	109 ± 14	107.7 ± 8.2	95.0 ± 2.8	30.2 ± 6.2	4.63 ± 0.88	32.3 ± 1.2	35.1 ± 2.8	13.96 ± 0.88	26.8 ± 2.1
**S16**	A	103 ± 11	102 ± 12	109.5 ± 8.3	101.6 ± 3.0	< 8.5	4.92 ± 0.80	50.5 ± 1.8	52.3 ± 4.2	21.0 ± 1.3	40.8 ± 3.2
	B	104 ± 12	105 ± 13	109.7 ± 8.3	104.2 ± 3.1	< 11.5	< 3.5	49.7 ± 1.8	52.5 ± 4.2	20.8 ± 1.3	40.7 ± 3.2
**S17**	A	33.3 ± 4.8	37.0 ± 6.3	31.7 ± 2.6	27.9 ± 1.2	< 9.4	< 3.0	17.9 ± 1.3	17.9 ± 1.6	7.17 ± 0.64	20.7 ± 3.5
	B	34.6 ± 5.6	17.0 ± 6.8	22.6 ± 1.8	22.8 ± 1.0	< 13.6	< 3.9	11.73 ± 0.70	12.6 ± 1.0	5.32 ± 0.44	28.1 ± 3.0

Uncertainties are expressed for a coverage factor of k=2.

Table S7. Activity concentration for the gamma emitters in the naturally occurring ^{238}U, ^{235}U, ^{232}Th and ^{40}K series, including ^{241}Am, in Slags.

**Series**			**^{238}U Radiactive Serie**					**^{235}U**	**^{232}Th Radiactive Serie**			**^{40}K**	**^{241}Am**
**Slags**			**^{234}Th**	**^{226}Ra**	**^{214}Pb**	**^{214}Bi**	**^{210}Pb**		**^{228}Ac**	**^{212}Pb**	**^{208}Tl**		
**S3**	Vitreous	A	44 ± 12	34 ± 11	39.1 ± 6.1	37.6 ± 2.8	30 ± 10	2.23 ± 0.61	62.5 ± 4.6	64 ± 10	23.8 ± 3.0	846 ± 73	3.26 ± 0.38
	Slag	B	35.3 ± 7.4	31.7 ± 7.8	32.7 ± 5.0	29.4 ± 1.9	26.5 ± 8.3	-	52.0 ± 3.5	53.6 ± 8.7	19.6 ± 2.4	632 ± 54	3.15 ± 0.35
**S4**	Steel	A	20.5 ± 5.3	22.6 ± 8.1	16.1 ± 2.6	15.0 ± 1.4	-	-	5.11 ± 0.61	5.7 ± 1.0	14.3 ± 2.9	-	4.06 ± 0.43
	Slag	B	15.3 ± 3.7	23.2 ± 5.0	17.8 ± 2.8	16.6 ± 1.5	-	-	5.53 ± 0.83	5.5 ± 1.0	49.4 ± 6.5	4.0 ± 2.9	3.77 ± 0.42

Uncertainties are expressed for a coverage factor of k=2.

Table S8. Chemical composition (wt. %) of the analysed Cements, Fly Ashe and Slag used for the models validations.

	**SiO_2**	**CaO**	**Al_2O_3**	**Fe_2O_3**	**MgO**	**MnO**	**Na_2O**	**K_2O**	**TiO_2**	**SO_3**	**LoI***
**OPC – CEM I 42.5 R**	20.00	62.90	4.20	4.00	1.20	0.00	0.00	0.80	0.20	2.62	3.40
**White Cement – BL I 52.5 R**	19.66	68.25	4.65	0.25	1.02	0.00	0.00	0.72	0.07	2.67	2.62
**White Cement – BL II/B-LL 42.5 R**	15.94	65.06	3.84	0.22	0.56	0.00	0.00	0.41	0.08	2.52	11.29
**CAC**	3.42	35.66	41.38	15.41	0.65	0.00	0.00	0.05	1.91	0.01	2.01
**CAC**	3.42	35.66	41.38	15.41	0.65	0.00	0.00	0.05	1.91	0.01	2.01
**Fly Ash**	52.86	3.70	22.07	8.21	2.28	0.07	1.15	2.53	1.04	0.10	4.92
**Slag**	34.46	42.08	11.47	0.61	8.10	0.27	0.00	0.45	0.80	1.68	0.00

**LoI: Lost on Ignition*

Table S9. Experimental Activity concentration for the gamma emitters in the naturally occurring ^{238}U, ^{235}U, ^{232}Th and ^{40}K series, in Cements, Fly Ash and Slag used for the models validations.

Series	^{238}U Radiactive Serie					^{235}U	^{232}Th Radiactive Serie			^{40}K
Validations	^{234}Th	^{226}Ra	^{214}Pb	^{214}Bi	^{210}Pb		^{228}Ac	^{212}Pb	^{208}Tl	
OPC CEM I 42.5 R	31.5 ± 4.7	31.6 ± 5.9	31.5 ± 2.5	30.1 ± 1.3	35.3 ± 6.2	< 4.2	16.59 ± 0.88	16.3 ± 1.3	6.03 ± 0.48	210 ± 10
White Cement BL I 52.5 R	30.4 ± 3.0	36.3 ± 4.6	32.0 ± 1.3	31.30 ± 0.78	35.4 ± 3.5	< 5.7	13.02 ± 0.57	10.84 ± 0.47	19.0 ± 1.1	220.8 ± 5.7
White Cement BL II/B-LL 42.5 R	25.3 ± 2.4	23.4 ± 3.2	23.19 ± 0.91	20.01 ± 0.43	33.4 ± 3.4	< 3.1	6.21 ± 0.23	8.54 ± 0.35	14.61 ± 0.58	94.5 ± 2.5
CAC	70.7 ± 3.9	70.6 ± 4.3	72.3 ± 2.7	69.2 ± 1.0	37.2 ± 3.0	2.69 ± 0.25	140.8 ± 2.3	146.7 ± 5.9	52.3 ± 1.6	< 3.0
CAC	63.7 ± 3.6	63.7 ± 4.0	58.7 ± 2.2	60.57 ± 0.86	29.7 ± 2.5	< 1.3	124.0 ± 2.0	110.8 ± 4.5	45.5 ± 1.4	< 4.2
Fly Ash	89.6 ± 4.9	91.2 ± 5.4	86.4 ± 3.3	86.6 ± 1.2	87.9 ± 6.5	< 1.5	64.1 ± 1.1	57.5 ± 2.3	23.02 ± 0.70	438.9 ± 9.4
Slag	115.6 ± 6.1	112.0 ± 6.6	120.6 ± 4.6	113.8 ± 1.7	18.9 ± 1.7	4.02 ± 0.22	43.56 ± 0.79	44.7 ± 1.8	16.44 ± 0.52	119.0 ± 2.8

Uncertainties are expressed for a coverage factor of k=2.

Annex 2

Collinearity in a multiple linear regression model is a common statistical problem that occurs when one or more independent variables in the model are a linear combination of one another, that is, there is a relationship between them.

A correlation matrix was used to determine the relationship between the independent variables (composition, wt.%) of cement, fly ash, and slag (A2.1, A2.2 and A2.3 Tables). The correlation between two variables is higher the closer their absolute values are near to 1.

Table S10. Correlation matrix between independent variables (wt. %) for Cements.

	SiO_2	CaO	Al_2O_3	Fe_2O_3	MgO	MnO	Na_2O	K_2O	TiO_2	SO_3
SiO_2	1.00									
CaO	0.18	1.00								
Al_2O_3	-0.71	-0.81	1.00							
Fe_2O_3	-0.62	-0.73	0.92	1.00						
MgO	0.34	-0.09	-0.24	-0.43	1.00					
MnO	0.04	0.22	-0.16	-0.22	0.18	1.00				
Na_2O	0.51	-0.37	-0.07	-0.21	0.64	0.49	1.00			
K_2O	0.91	0.00	-0.54	-0.51	0.36	-0.11	0.55	1.00		
TiO_2	-0.58	-0.80	0.94	0.97	-0.32	-0.21	-0.11	-0.44	1.00	
SO_3	0.11	0.05	-0.23	-0.53	0.72	0.06	0.37	0.17	-0.44	1.00

Table S11. Correlation matrix between independent variables (wt. %) for Fly Ashes.

	SiO_2	CaO	Al_2O_3	Fe_2O_3	MgO	MnO	Na_2O	K_2O	TiO_2	SO_3
SiO_2	1.00									
CaO	-0.43	1.00								
Al_2O_3	-0.22	-0.44	1.00							
Fe_2O_3	-0.56	0.11	-0.27	1.00						
MgO	-0.09	0.51	-0.67	0.01	1.00					
MnO	0.11	0.51	-0.20	-0.08	-0.12	1.00				
Na_2O	0.21	0.49	-0.47	-0.31	0.51	0.43	1.00			
K_2O	0.54	-0.13	-0.45	-0.10	0.21	-0.02	0.24	1.00		
TiO_2	-0.13	-0.19	0.51	-0.27	-0.29	-0.17	-0.57	-0.41	1.00	
SO_3	-0.20	0.28	-0.01	0.27	-0.23	0.60	0.06	-0.27	0.03	1.00

Table S12. Correlation matrix between independent variables (wt. %) for Slags.

	SiO_2	Al_2O_3	CaO	MgO	Na_2O	K_2O	TiO_2
SiO_2	1.00						
Al_2O_3	-0.29	1.00					
CaO	-0.59	0.10	1.00				
MgO	0.41	-0.75	-0.23	1.00			
Na_2O	0.12	0.16	-0.16	-0.30	1.00		
K_2O	0.25	0.59	-0.25	-0.55	0.66	1.00	
TiO_2	-0.11	0.13	0.53	-0.24	-0.17	0.01	1.00

Due to the collinearity issues that arise from introducing each and every variable when constructing the linear regression models in this work, we chose the backward stepwise regression methodology, in which a model is obtained by iteratively discarding the variables with values of $\alpha > 0.05$ until a model with independent variables with P-values less than 0.05 is obtained.

After obtaining the multiple linear regression models for ^{226}Ra, ^{232}Th (^{212}Pb), and ^{40}K for cements, fly ash, and slag, the impact of collinearity in the variables maintained in each of the models was investigated. The variance inflation factor (VIF) (A2.4), was calculated to quantify the intensity of the variables collinearity, using the following expression:

$$VIF = \frac{1}{1 - R_i^2}$$

Where R^2_i is the auxiliary coefficient of determination for each independent variable maintained in each model.

Table S13. Calculated VIF for the variables in the ^{226}Ra, ^{232}Th (^{212}Pb) and ^{40}K models for cements, Fly Ash and Slags.

Cements

^{226}Ra	SiO_2	Fe_2O_3	MnO	TiO_2
R^2_i	0.33	0.97	0.10	0.97
VIF	1.50	35.55	1.11	33.60

^{212}Pb	CaO	Al_2O_3	MgO	K_2O	TiO_2	SO_3
R^2_i	0.77	0.97	0.91	0.77	0.97	0.86
VIF	4.31	34.25	11.45	4.38	34.96	7.00

^{40}K	SiO_2	CaO	Al_2O_3	Na_2O	K_2O	TiO_2
R^2_i	0.98	0.93	0.97	0.73	0.96	0.96
VIF	52.37	13.56	30.45	3.69	23.08	25.48

Fly Ash (FA)

^{226}Ra	Al_2O_3	Fe_2O_3	MgO	TiO_2
R^2_i	0.93	0.63	0.64	0.92
VIF	13.42	2.67	2.80	13.23

^{212}Pb	CaO	Al_2O_3	Fe_2O_3	MgO
R^2_i	0.80	0.68	0.64	0.78
VIF	4.91	3.16	2.77	4.53

^{40}K	K_2O	TiO_2
R^2_i	0.66	0.66
VIF	2.99	2.99

Slag (S)

^{226}Ra	Na_2O	K_2O	TiO_2
R^2_i	0.54	0.70	0.46
VIF	2.18	3.39	1.87

^{212}Pb	CaO	Na_2O	K_2O
R^2_i	0.43	0.51	0.67
VIF	1.76	2.04	3.06

^{40}K	SiO_2	CaO	MgO	TiO_2
R^2_i	0.88	0.88	0.83	0.84
VIF	8.51	8.10	6.01	6.37

According to Kleinbaum et al., [87], VIF values greater than 10 indicate collinearity issues that are aggravated depending on the magnitude of this value. Collinearity has been minimised in the current work using the backward stepwise regression model, which has very little impact on our models because these variables are vector-correlated, as shown in the HJ-Biplot plots in the manuscript.
